# Chemoprophylactic Assessment of Combined Intranasal SARS-CoV-2 Polymerase and Exonuclease Inhibition in Syrian Golden Hamsters

**DOI:** 10.3390/v15112161

**Published:** 2023-10-27

**Authors:** Eduardo Gallardo-Toledo, Megan Neary, Joanne Sharp, Joanne Herriott, Edyta Kijak, Chloe Bramwell, Paul Curley, Usman Arshad, Henry Pertinez, Rajith K. R. Rajoli, Anthony Valentijn, Helen Cox, Lee Tatham, Anja Kipar, James P. Stewart, Andrew Owen

**Affiliations:** 1 Department of Pharmacology and Therapeutics, Institute of Systems, Molecular and Integrative Biology, University of Liverpool, Liverpool L69 3BX, UK; e.gallardo@liverpool.ac.uk (E.G.-T.); flanders@liverpool.ac.uk (J.H.); e.kijak@liverpool.ac.uk (E.K.); c.bramwell@liverpool.ac.uk (C.B.); coxy67@liverpool.ac.uk (H.C.); l.tatham@liverpool.ac.uk (L.T.); 2 Centre of Excellence in Long-Acting Therapeutics (CELT), University of Liverpool, Liverpool L69 3BX, UK; 3 Department of Infection Biology & Microbiomes, Institute of Infection, Veterinary and Ecological Sciences, University of Liverpool, Liverpool L69 3BX, UK; 4 Laboratory for Animal Model Pathology, Institute of Veterinary Pathology, Vetsuisse Faculty, University of Zurich, 8057 Zurich, Switzerland

**Keywords:** SARS-CoV-2, pibrentasvir, favipiravir, remdesivir, chemoprophylaxis

## Abstract

Pibrentasvir (PIB) has been demonstrated to block exonuclease activity of the SARS-CoV-2 polymerase, protecting favipiravir (FVP) and remdesivir (RDV) from post-incorporation excision and eliciting antiviral synergy in vitro. The present study investigated the chemoprophylactic efficacy of PIB, FVP, RDV, FVP with PIB, or RDV with PIB dosed intranasally twice a day, using a Syrian golden hamster contact transmission model. Compared to the saline control, viral RNA levels were significantly lower in throat swabs in FVP (day 7), RDV (day 3, 5, 7), and RDV+PIB (day 3, 5) treatment groups. Similarly, findings were evident for nasal turbinate after PIB and RDV treatment, and lungs after PIB, FVP, and FVP+PIB treatment at day 7. Lung viral RNA levels after RDV and RDV+PIB treatment were only detectable in two animals per group, but the overall difference was not statistically significant. In situ examination of the lungs confirmed SARS-CoV-2 infection in all animals, except for one in each of the RDV and RDV+PIB treatment groups, which tested negative in all virus detection approaches. Overall, prevention of transmission was observed in most animals treated with RDV, while other agents reduced the viral load following contact transmission. No benefit of combining FVP or RDV with PIB was observed.

## 1. Introduction

While the World Health Organization (WHO) has declared the pandemic over, there is still concern about the possible appearance of new variants of SARS-CoV-2 in the future [1]. While vaccines [2], antiviral [3] and diseases-modifying drugs [4], as well as monoclonal antibodies [5] were assessed and deployed successfully, continued assessment of putative antiviral interventions is warranted to prepare for unpredictable future outbreaks.

Several repurposed drugs have been studied for prophylaxis and/or treatment of SARS-CoV-2 infection, but many were studied without a clear understanding of the mechanism of action or the plausibility of the pharmacokinetic–pharmacodynamic relationship [4,6,7]. Where the mechanism of action is understood, viral targets are involved in entry mechanisms (via viral spike, angiotensin-converting enzyme 2, endosomal entry, or transmembrane protease serine 2), RNA replication and transcription (via the RNA-dependent RNA polymerase [RdRp]), or maturation (via the viral protease) [8]. Favipiravir (FVP) is a nucleotide analogue that is incorporated into the replicated RNA by the viral RdRp and elicits antiviral efficacy via lethal mutagenesis [9]. Conversely, while remdesivir (RDV) is also a nucleotide analogue, it elicits antiviral efficacy via delayed chain termination [10]. RDV is conditionally recommended by WHO for use in patients with non-severe COVID-19 at the highest risk of hospitalisation [11]. Insufficient randomised controlled trial evidence currently exists for FVP, but it has been used off label in some regions.

Drug combinations have proven to be successful across a raft of viruses and infectious diseases, including human immunodeficiency virus (HIV), hepatitis C virus (HCV), tuberculosis, and malaria [12,13,14]. Combinations of anti-infective agents have a number of explicit advantages, including increased efficacy in comparison to monotherapies and mitigation of the rate at which resistance emerges. The utilisation of antiviral drug combinations for treatment and prevention of COVID-19 has already been proposed [15,16], but the speed at which antiviral development was needed complicated the rate at which they could be assessed. In broad terms, drug combinations can be segregated into three categories: (1) combination of two or more antiviral drugs, (2) combination of an antiviral drug with a pharmacoenhancer, or (3) combination between antiviral(s) and disease modifying agents. The current work focuses on assessment of a combination that may fall into one of the first two categories.

Recent research has demonstrated that exonuclease activity of the SARS-CoV-2 RdRp is able to excise FVP and RDV from the viral RNA after incorporation, which reduces the in vitro potency of these drugs [17]. Accordingly, Wang and coauthors hypothesised that inclusion of an exonuclease inhibitor would increase the antiviral activity of FVP and RDV, and demonstrated that pibrentasvir (PIB), a non-structural protein 5A (NS5A) inhibitor of HCV replication, blocks SARS-CoV-2 exonuclease and synergises with FVP and RDV in vitro [18]. These data present a solid basis for further evaluation of the potential utility of combined polymerase and exonuclease inhibition in vivo. Syrian golden hamsters are a well-established model of SARS-CoV-2 respiratory infection [19], which has been proven useful for the investigation of chemoprophylactic agents in SARS-CoV-2 transmission studies [20,21,22,23,24]. Intranasal administration deployed in a transmission model offers the opportunity to maximise drug concentrations at the site and time of infection, thereby minimising the bar for demonstrating in vivo antiviral potential.

This work sought to investigate the in vivo antiviral efficacy for combinations of PIB with FVP or RDV following intranasal delivery in a Syrian golden hamster transmission model. The presented data demonstrate that transmission of SARS-CoV-2 can be blocked by intranasal delivery of antiviral drugs. However, no evidence of a synergistic antiviral effect was observed when PIB was combined with either FVP or RDV.

## 2. Materials and Methods

### 2.1. Animals and Materials

Male Syrian golden hamsters (*Mesocricetus auratus*) were purchased from Janvier Labs (Essex, UK). PIB, FVP, and RDV were purchased from BIOSYNTH LTD (Newbury, UK). An amount of 1 mL regular flocked swabs was obtained from Appleton Woods (Birmingham, UK). Precellys CKmix lysing tubes and a bead mill homogeniser were purchased from Bertin Technologies (Montigny-le-Bretonneux, France) and Fisher Scientific (Waltham, MA, USA), respectively. SARS-CoV-2 (2019-nCoV) CDC qPCR Probe Assay and CDC RUO 2019-nCoV_N_Positive Control were purchased from IDT (Newark, NJ, USA). GoTaq^®^ Probe 1-Step RT-qPCR System and qTOWER^3^ Real-Time PCR Detector were purchased from Promega (Fitchburg, WI, USA) and Analytik Jena (Jena, Germany), respectively. TRIzol reagent, GlycoBlue^TM^, Phasemaker^TM^ tubes, Nanodrop, and TURBO DNA-free^TM^ kits were purchased from Thermo Fisher (Waltham, MA, USA). For immunohistology, the rabbit anti-SARS-CoV nucleoprotein antibody was purchased from Rocklands (Pottstown, PA, USA), the peroxidase blocking buffer and the Envision+System HRP Rabbit from Agilent (Carpinteria, CA, USA), the diaminobenzidine from Agilent DAKO (Carpinteria, CA, USA), and the Tissue-Tek Film for coverslipping from Sysmex (Hyogo, Japan).

### 2.2. Virus Isolation

Human nCoV19 isolate/England/202012/01B (lineage B.1.1.7 Alpha variant) was obtained from the National Infection Service at Public Health England, Porton Down, UK, via the European Virus Archive (catalogue code 004V-04032). This was supported by the European Virus Archive GLOBAL (EVA-GLOBAL) project that has received funding from the European Union’s Horizon 2020 research and innovation programme under grant agreement No. 871029.

### 2.3. Ethical Approval

All animal experiments were carried out under the UK Home Office Project License PP4715265 in accordance with the UK Home Office Animals Scientific Procedures Act (ASPA 1986) and approved by the local University of Liverpool Animal Welfare and Ethical Review Body. All procedures involving SARS-CoV-2 were conducted at a containment level 3 (CL3) facility and approved by the UK Health and Safety Executive and the University of Liverpool Biohazards Sub-Committee.

### 2.4. SARS-CoV-2 Transmission Model in Syrian Golden Hamsters

Hamsters (male, 80–100 g) were housed in ventilated cages with environmental enrichment at 21 ± 2 °C, 12 h light/dark cycle, and food and water ad libitum. Animals were weighed and monitored daily for the duration of the study.

**Figure 1:**
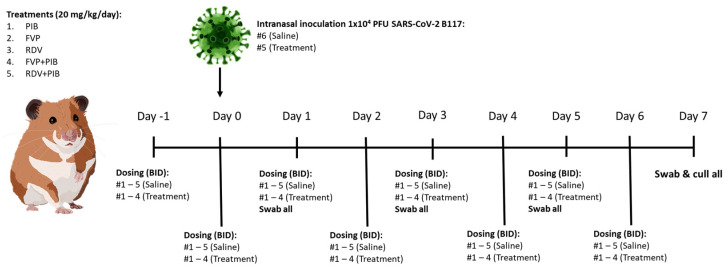
Study design for evaluation of the chemoprophylactic efficacy of pibrentasvir (PIB), favipiravir (FVP), remdesivir (RDV), FVP+PIB, or RDV+PIB dosed intranasally twice a day (BID); 10 mg/kg BID, total 20 mg/kg/day. The control group was dosed with saline. Treatments started 24 h prior to being co-housed with an untreated hamster intranasally inoculated with 1 × 10^4^ PFU SARS-CoV-2 B117. All animals were throat swabbed at day 1, 3, 5, and 7.

Hamsters were randomly divided into 6 treatment groups (n = 5, control group n = 6). All but one animal in each group were intranasally dosed twice a day (BID) with saline (control; saline #1−5) or 20 mg/kg/day of PIB (PIB #1−4), FVP (FVP #1−4), RDV (RDV #1−4), FVP with PIB (FVP+PIB #1−4), or RDV with PIB (RDV+PIB #1−4) from day −1 to 6 (Figure 1). At day 0, the six untreated animals were anaesthetised (3% isoflurane) and intranasally infected with SARS-CoV-2 B117 (1 × 10^4^ PFU/hamster in 100 µL PBS; infected #1−6) and then each one introduced into the cages of the different treatment groups, where they were co-housed in Tecniplast GR1800 Double Decker cages with one infected and the treated naïve hamsters to allow contact transmission. All hamsters were weighed daily throughout the study from day −1 to 7 days post infection (dpi). All hamsters were swabbed (throat) at 1, 3, 5, and 7 dpi. They were culled on day 7 via intraperitoneal injection of pentobarbitone. Samples from the right lung and nasal turbinates were then harvested and stored at −80 °C for qRT-PCR analysis. Left lungs were collected and fixed in 10% buffered formalin for histological analysis.

### 2.5. Quantitative RT-PCR Analysis of Throat Swabs and Tissue Samples

Throat swabs and tissue samples from right lungs and nasal turbinates were inactivated in a Class II Biosafety cabinet using TRIzol LS or TRIzol, respectively. Then, 260 µL of swab buffer was transferred into 2 mL screw-cap vials and mixed with 750 µL of TRIzol LS reagent (ThermoFisher, Runcorn, UK). Lung or nasal turbinate material was transferred into Precellys CKmix lysing tubes (Bertin Technologies); mixed with 1 mL TRIzol reagent (ThermoFisher, Runcorn, UK); and homogenised for two cycles at 3.55 m per second, for 30 s, using a bead mill homogeniser (Fisher Scientific, Loughborough, UK). Samples were stored at −80 °C until further analysis.

For RNA extraction, inactivated samples were thawed and transferred to Phasemaker^TM^ tubes (ThermoFisher, Runcorn, UK), mixed with 200 µL chloroform, and processed according to the manufacturer’s instructions. The aqueous phase was transferred to new tubes, mixed with 500 µL isopropanol, and precipitated using GlycoBlue^TM^ (ThermoFisher, Runcorn, UK) following the manufacturer’s instructions. Samples were solubilised in RNAase-free water and the RNA quantified using a Nanodrop (ThermoFisher, Runcorn, UK). Subsequently, the RNA samples were DNAse treated using the TURBO DNA-free^TM^ kit following the manufacturer’s instructions and stored at −80 °C until the qRT-PCR was performed.

Total (N-gene) viral RNA was quantified by quantitative (q)RT-PCR, following a protocol adapted from the CDC 2019-Novel Coronavirus (2019-nCoV) Real-Time PCR Diagnostic Panel [25]. Briefly, a tenfold serial dilution of CDC RUO 2019-nCoV_N_Positive Control (IDT) and 18S positive control were used to prepare standard curves of 200,000−2 copies/reaction and 5 × 10^8^−50 copies/reaction, respectively. Methods for the generation of the 18S standards have been outlined previously [26]. A reaction master mix was prepared using the GoTaq^®^ Probe 1-Step RT-qPCR System (Promega), N1 primer/probe mix from the SARS-CoV-2 (2019-nCoV) CDC qPCR Probe Assay (IDT), and 18S RNA primers (300 nM) and probe (200 nM) sequences previously reported (IDT) [27].

The qPCR standards, DNAse-treated RNA (200 ng/mL), or nuclease-free water (negative control) were mixed with the reaction master mix (20 µL final volume) in respective wells and run using a qTOWER^3^ Real-Time PCR Detector (Analytik Jena). The thermal cycling conditions for the qRT-PCR reactions were as follows: 1 cycle of 45 °C for 15 min, 1 cycle of 95 °C for 2 min, 40 cycles of 95 °C for 3 s, and 55 °C for 30 s. The N1-RNA data were subsequently normalised to 18S data for viral RNA quantification. The limit of detection (LOD) for the assay was defined as a N1-RNA value of ≤2 copies/reaction and a PCR Ct value cut-off ≥32 cycles, in accordance with previously published data [28,29].

### 2.6. Histological and Immunohistological Analyses

The left lungs from each animal were fixed in 10% buffered formalin for 48 h and then stored in 70% ethanol until further processing. Two longitudinal sections were prepared from each lung and routinely paraffin wax embedded. Consecutive sections (3–5 µm) were prepared and stained with haematoxylin eosin (HE) for histological examination or subjected to immunohistological staining to detect SARS-CoV-2 antigen (performed in an autostainer; Agilent), using the horseradish peroxidase (HRP) method and rabbit anti-SARS-CoV nucleocapsid protein (Rockland) as previously described [24,30].

### 2.7. Statistical Analysis

Statistical significance for viral RNA load in throat swabs, nasal turbinate, and lung samples was determined using a nonparametric Mann–Whitney test (one-tailed). A 2-way ANOVA multiple comparison with Bonferroni correction was applied to compare the percentage weight change. Statistical significance was determined by *p* ≤ 0.05. Statistical analysis was calculated using GraphPad Prism (v 10.0.2).

## 3. Results

### 3.1. Changes in Body Weight

**Figure 2:**
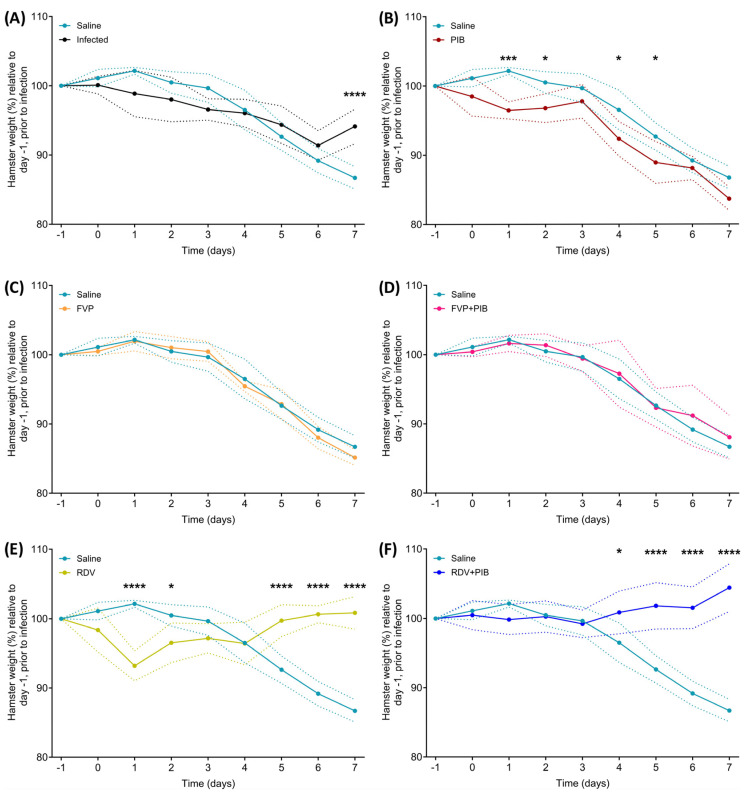
Hamsters from each group (n = 4, except saline n = 5) and infected hamsters (n = 6) were weighed daily from day −1 to day 7 post infection. All weights are shown as a percentage of the initial weight recorded at baseline, day −1 of the study. (**A**) Saline vs. infected, (**B**) Saline vs. PIB, (**C**) Saline vs. FVP, (**D**) Saline vs. FVP with PIB, (**E**) Saline vs. RDV, (**F**) Saline vs. RDV with PIB. Statistical significance was determined by 2-way ANOVA multiple comparison with Bonferroni correction. * = *p* ≤ 0.05, *** = *p* ≤ 0.001, **** = *p* ≤ 0.0001. Dotted lines represent standard deviations.

In order to follow the clinical course of the disease, all animals were weighed daily throughout the study (−1 to 7 dpi). The average weight at each time point was expressed as the percentage relative to the average weight at day −1 prior to infection (Figure 2). Intranasally infected hamsters lost weight progressively from day 1 to day 6 (average 8.6% weight loss). By 7 dpi, animals regained some weight (average 5.9% weight loss; Figure 2A). Body weight changes were concordant with those previously reported for hamsters after intranasal SARS-CoV-2 infection [31,32]. In the saline-treated control group, loss of weight was first observed on day 3, and the weight decreased progressively until day 7 (average 13.3% weight loss; Figure 2A).

Weight loss of hamsters treated with PIB began at day 0; the weight remained steady until day 3, but then progressively decreased until day 7 (average 16.3% weight loss). Body weights were significantly lower than the weights of the saline-treated controls on day 1 (*p* ≤ 0.001), day 2 (*p* ≤ 0.05), day 4 (*p* ≤ 0.05), and day 5 (*p* ≤ 0.05; Figure 2B). Treatment groups receiving FVP or FVP with PIB lost weight from day 4 onwards, which progressively decreased until day 7 (average 14.8% and 11.9% weight loss, respectively) and similar to that of the saline-treated control group (Figure 2C,D). The RDV treatment group displayed weight loss at day 0, and weights were significantly lower than those of the saline-treated controls on day 1 (*p* ≤ 0.0001) and day 2 (*p* ≤ 0.05; Figure 2E). However, animals regained weight between day 1 and day 2, which continued to progressively increase and was significantly higher than in the saline-treated controls group by day 7 (*p* ≤ 0.0001).

The RDV with PIB treatment group showed no weight loss throughout the study (Figure 2F) and increased in body weight from day 4 onwards, being significantly higher than the weight of the saline-treated controls from day 5 onwards (*p* ≤ 0.0001; Figure 2F).

### 3.2. Viral RNA Quantification in Throat Swabs and Tissue Samples

**Figure 3:**
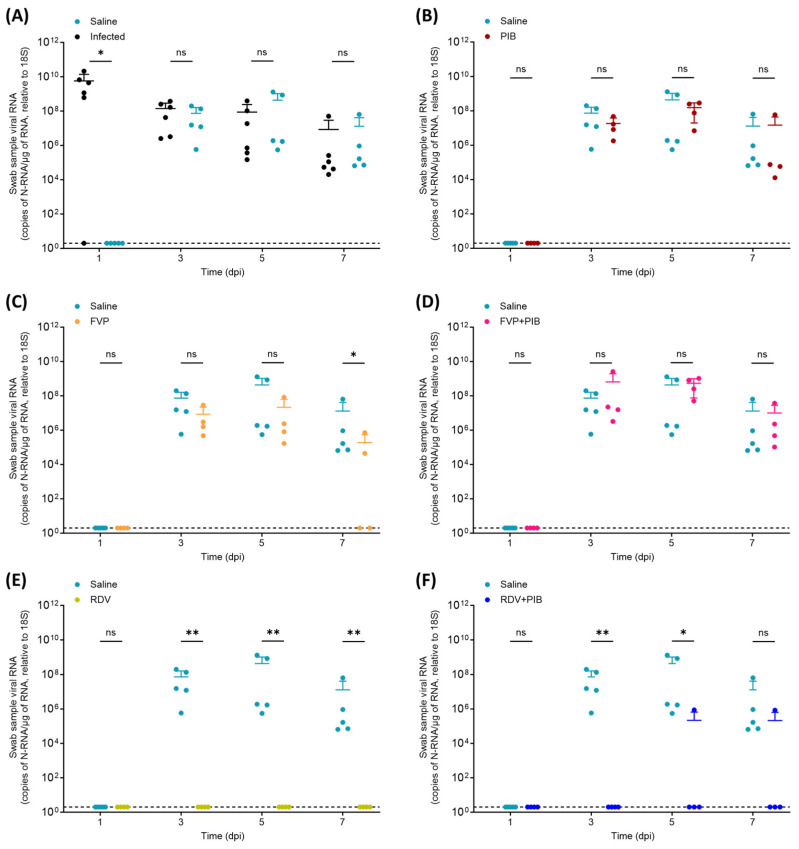
Quantification of SARS-CoV-2 N-RNA in throat swabs taken on day 1, 3, 5, and 7 post intranasal inoculation of the donor (i.e., infected) hamsters. (**A**) Saline vs. Infected, (**B**) Saline vs. PIB, (**C**) Saline vs. FVP, (**D**) Saline vs. FVP+PIB, (**E**) Saline vs. RDV, (**F**) Saline vs. RDV+PIB. To determine statistical significance (*p* ≤ 0.05), a nonparametric Mann–Whitney test (one-tailed) was performed between the saline control treated group (n = 5) and the infected group (n = 6) or the corresponding chemoprophylaxis-treated group (n = 4) for each day. ns = not statistically significant, * = *p* ≤ 0.05, ** = *p* ≤ 0.01. LOD: limit of detection (indicated by dotted line).

**Figure 4:**
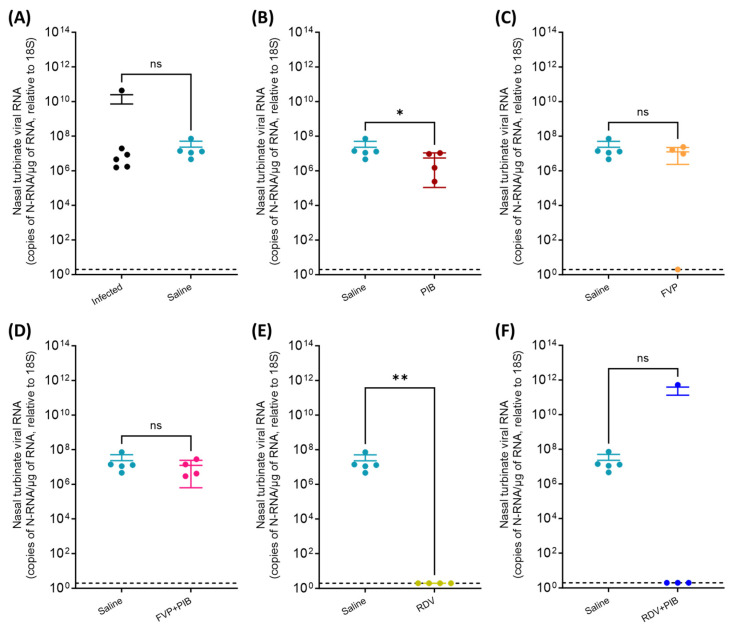
Quantification of SARS-CoV-2 N-RNA in nasal turbinate samples at day 7 post intranasal inoculation of the donor (i.e., infected) hamsters. (**A**) Saline vs. Infected, (**B**) Saline vs. PIB, (**C**) Saline vs. FVP, (**D**) Saline vs. FVP+PIB, (**E**) Saline vs. RDV, (**F**) Saline vs. RDV+PIB. Comparison between saline control group (n = 5) and the infected group (n = 6) or the corresponding chemoprophylaxis-treated group (n = 4) was performed to determine statistical significance (nonparametric Mann–Whitney test [one-tailed], *p* ≤ 0.05). ns = not statistically significant, * = *p* ≤ 0.05, ** = *p* ≤ 0.01. LOD: limit of detection (indicated by dotted line).

**Figure 5:**
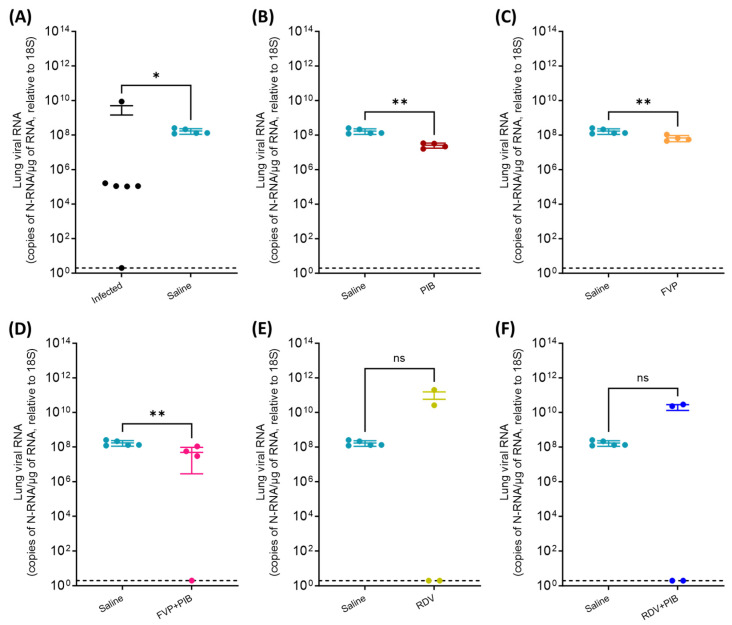
Quantification of SARS-CoV-2 N-RNA in lung samples at day 7 post intranasal inoculation of the donor (i.e., infected) hamsters. Comparison between saline control group (n = 5) and treatment groups (n = 4). (**A**) Saline vs. Infected, (**B**) Saline vs. PIB, (**C**) Saline vs. FVP, (**D**) Saline vs. FVP+PIB, (**E**) Saline vs. RDV, (**F**) Saline vs. RDV+PIB. Statistical significance (*p* ≤ 0.05) was determined using a nonparametric Mann–Whitney test (one-tailed) between the saline control group (n = 5) and the infected group (n = 6) or the corresponding chemoprophylaxis-treated group (n = 4). ns = not statistically significant, * = *p* ≤ 0.05, ** = *p* ≤ 0.01. LOD: limit of detection (indicated by dotted line).

Viral N-RNA levels in throat swabs, nasal turbinate, and lung samples are shown in Figure 3, Figure 4 and Figure 5, respectively. Throat swabs were taken at day 1, 3, 5, and 7 post intranasal infection of the six donor hamsters. In directly infected hamsters, viral N-RNA was detected in the swabs from day 1 to day 7, except in the swab sample of infected animal #5 at day 1, where viral RNA was below the LOD (Figure 3A). This confirms that the animals were consistently shedding virus from day 1 onwards. In hamsters exposed to virus shed from the infected hamsters, viral RNA was undetectable in swabs on day 1, but then consistently detected on days 3, 5, and 7 in the saline-treated, as well as the PIB- and FVP-with-PIB-treated, hamsters (Figure 3A,B,D). In saline-treated hamsters, the viral RNA in swabs was similar to the amount in infected hamsters on days 3, 5, and 7, with a tendency for being higher on day 5 (Figure 3A). In PIB- and FVP-with-PIB-treated animals, no significant difference in viral RNA in swabs compared to saline-treated hamsters was observed at any of the three time points (Figure 3B,D). In the FVP-treated animals, viral RNA in swabs was generally lower than in the saline-treated controls. Also, at day 7, viral N-RNA in swabs was below the LOD in two animals and, overall, significantly lower than in the saline control group (1.3 × 10^7^ vs. 1.9 × 10^5^ copies of N-RNA/µg of RNA relative to 18S; *p* = 0.048; Figure 3C). In the RDV-treated group, no hamster showed detectable levels of viral RNA in swabs during the 7 days (Figure 3E). For the RDV with PIB treatment, swabs were negative at day 3, whereas at the other time points, one animal each exhibited a low viral RNA level (day 5: #2; day 7: #3; Figure 3F). For both the RDV and RDV with PIB groups, the viral RNA in swabs was significantly lower than in the saline control group at day 3 (7.3 × 10^7^ vs. <LOD copies of N-RNA/µg of RNA relative to 18S; *p* = 0.008 for both comparisons) and day 5 (4.3 × 10^8^ vs. <LOD copies of N-RNA/µg of RNA relative to 18S, *p* = 0.008; and 4.3 × 10^8^ vs. 2.2 × 10^5^ copies of N-RNA/µg of RNA relative to 18S, *p* = 0.016, respectively). At day 7, the difference was only significant for the RDV treatment group (1.3 × 10^7^ vs. <LOD copies of N-RNA/µg of RNA relative to 18S, *p* = 0.008).

At termination on day 7, viral N-RNA was detected in the nasal turbinate from all infected hamsters, as well as the saline-, PIB-, and FVP-with-PIB-treated groups (Figure 4A,B,D), confirming infection of the upper respiratory tract (nasal mucosa). The FVP-treated animals had similar viral RNA levels in the nasal turbinate, except for one (animal #1), which was below the LOD (Figure 4C). All animals that had undergone RDV treatment were also negative for viral RNA (Figure 4E) in nasal turbinate, as were three animals in the RDV with PIB group (Figure 4F; animal #3, also tested positive in the throat swab on day 7). A significant difference in viral RNA in nasal turbinate was only observed for the PIB and the RDV treatment groups, with lower levels in both groups (2.3 × 10^7^ vs. 5.5 × 10^6^ copies of N-RNA/µg of RNA relative to 18S, *p* = 0.032; and 2.3 × 10^7^ vs. <LOD copies of N-RNA/µg of RNA relative to 18S, *p* = 0.008, respectively).

At 7 dpi, viral RNA was detected in the lungs of five of the six infected hamsters and all saline-treated hamsters, with significantly lower viral RNA in the latter (1.5 × 10^9^ vs. 1.7 × 10^8^ copies of N-RNA/µg of RNA relative to 18S, *p* = 0.037; Figure 5A). Viral N-RNA was also consistently detected in all PIB-, FVP-, and FVP-with-PIB-treated hamsters, apart from one animal in the latter group, where it was below the LOD (animal #3), and at significantly lower levels than in the saline-treated control group (1.7 × 10^8^ vs. 2.6 × 10^7^ copies of N-RNA/µg of RNA relative to 18S, *p* = 0.008; 1.7 × 10^8^ vs. 6.8 × 10^7^ copies of N-RNA/µg of RNA relative to 18S, *p* = 0.008; and 1.7 × 10^8^ vs. 4.9 × 10^7^ copies of N-RNA/µg of RNA relative to 18S, *p* = 0.008, respectively; Figure 5B–D). Interestingly, half of the animals (2/4) in both the RDV and RDV with PIB groups displayed viral N-RNA levels below the LOD.

Information on copies of N-RNA/µg of RNA relative to 18S in the different samples at day 7 in individual animals is provided in Supplementary Table S1, and information on average copies of N-RNA/µg in the different treatment groups, as well as the *p*-values obtained in the comparison with saline controls, are provided in Supplementary Table S2.

### 3.3. Pathological Changes and Viral Antigen Expression in the Lungs

**Figure 6:**
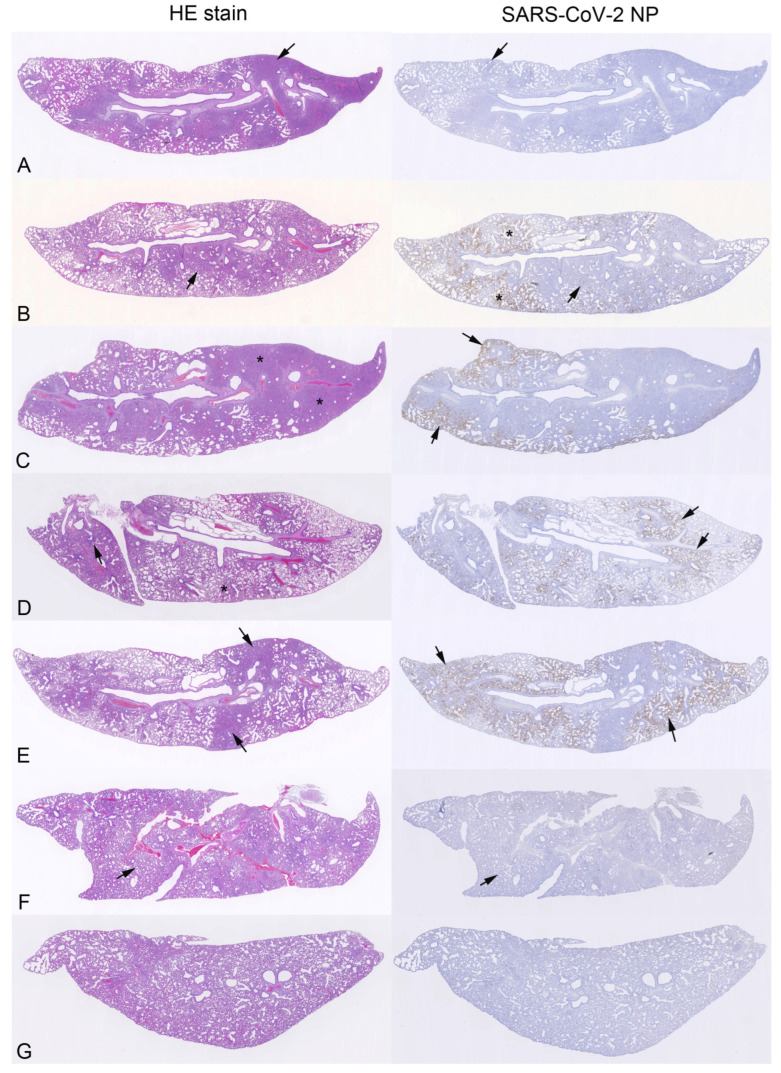
Histological changes and viral antigen expression in the lungs of hamsters intranasally infected with SARS-CoV-2 (**A**) or treated with saline (**B**), PIB (**C**), FVP (**D**), FVP with PIB (**E**), RDV (**F**), or RDV with PIB (**G**) and housed together as a group of 5 hamsters with an infected hamster; animals were euthanised and examined at day 7. Left column: HE-stained sections; right column: consecutive sections stain for SARS-CoV-2 nucleoprotein (NP), immunohistology, and haematoxylin counterstain. (**A**) Infected hamster (#3). The lung parenchyma exhibits multifocal-to-coalescing consolidated areas (arrow). Viral antigen expression (right image) is seen in alveolar epithelial cells (AECs) in consolidated areas and patches of unaltered alveoli. Arrows: area shown in higher magnification in Supplementary Figure S1A. (**B**) Saline-treated control animal (#4). The histological changes are dominated by multifocal areas of alveoli that contain desquamed AEC and/or alveolar macrophages (AMs) and exhibit activated type II pneumocytes, some syncytial cells and some degenerate cells. There is widespread viral antigen expression in AEC in large patches of alveoli (*) and, less extensively, in consolidated areas (arrow). Arrows: area shown in higher magnification in Supplementary Figure S1B. (**C**) PIB-treated hamster (#4). The lung parenchyma exhibits focal extensive consolidation (*). There is widespread viral antigen expression in AEC in large patches of alveoli (arrows). (**D**) FVP-treated hamster (#2). The lung parenchyma exhibits focal desquamative changes (*) and small focal consolidated areas with epithelial hyperplasia (arrow). There is widespread viral antigen expression in AEC in large patches of alveoli (arrows, right image). (**E**) FVP+PIB-treated animal (#2). The lung parenchyma exhibits focal consolidated areas with focal epithelial hyperplasia (arrows, left image). There is widespread viral antigen expression in AEC in large patches of alveoli (arrows, right image). (**F**) RDV-treated hamster (#1). The lung parenchyma appears cell rich (due to increased interstitial cellularity), but otherwise unaltered. There is no evidence of viral antigen expression (right image). Arrows: area shown in higher magnification in Supplementary Figure S1C. (**G**) RDV-with-PIB-treated hamster (#4). The lung parenchyma is widely unaltered, and there is no evidence of viral antigen expression.

The histological examination of the lungs in combination with the immunohistological staining for SARS-CoV-2 NP showed pathological changes consistent with SARS-CoV-2 infection at 7 dpi in this species [31,32,33] in all intranasally infected hamsters. Besides a variable extent of increased interstitial cellularity, these were represented by multifocal-to-coalescing consolidated areas that occupied a large proportion of the lung parenchyma and comprised focal hyperplasia of type II pneumocytes/bronchiolar epithelial cells (BEC), leukocyte infiltration (macrophages, fewer lymphocytes, and variable numbers of neutrophils), and some degenerate cells, with variable degree (Figure 6A, Supplementary Figure S1A). This was generally accompanied by some degree of perivascular lymphocyte-dominated infiltration and periarterial edema. Viral antigen expression was detected in all lungs, but was very limited and restricted to a few macrophages and alveolar epithelial cells (AECs) within consolidated areas and AECs in a few small patches of alveoli (Figure 6A, Supplementary Figure S1A). This is in agreement with the low viral RNA levels found in the lungs of all but one infected hamster (Supplementary Table S1).

In the saline-treated control animals, the histological changes were dominated by multifocal areas of alveoli with desquamed AEC/alveolar macrophages (AMs), with the presence of activated type II pneumocytes, some syncytial cells and some degenerate cells, often accompanied by alveolar edema (Figure 6B; Supplementary Figure S1B), all consistent with SARS-CoV-2 infection early after intranasal challenge in this species [31,32,33]. The changes were accompanied by widespread viral antigen expression, generally in multiple large patches of alveoli with positive AEC (Figure 6B; Supplementary Figure S1B). In some (3/5) animals, there was evidence of viral antigen in BEC, which was also occasionally found to be degenerate. Three animals also exhibited some consolidated areas with focal hyperplasia of type II pneumocytes/BEC, as seen in the infected hamsters. These findings indicate that the lungs were infected for at least 3 days (the hyperplastic epithelial changes can be seen as early as 3 dpi; ref. [33]), whereas the lungs with the purely desquamative changes were likely infected for a shorter time period.

The PIB-, FVP-, and FVP-with-PIB-treated animals all exhibited histological changes consistent with SARS-CoV-2 infection, and viral antigen expression confirmed infection of the lungs. In all three groups, both areas of alveoli with desquamed AEC/AM (the dominating change in the saline-treated control hamsters) and consolidated areas with focal hyperplasia of type II pneumocytes/BEC were observed. The latter were seen in all hamsters with PIB treatment, although these were generally less extensive than in the infected hamsters (Figure 6C) and accompanied in most animals (3/4) by the former desquamative changes. In contrast, the desquamative changes were present in all four FVP-treated hamsters, whereas the focal epithelial hyperplasia was only observed in two animals (Figure 6D). In all four FVP-with-PIB-treated animals, both types of changes were obvious (Figure 6E). There was generally widespread viral antigen expression (Figure 6C–E). Interestingly, the one FVP-with-PIB-treated animal in which the viral RNA level was below the LOD (animal #3) exhibited as widespread viral antigen expression as the other animals in this group (Supplementary Table S1). These findings indicate variable lengths of time for pulmonary infection in these groups of hamsters; the generally lesser extent of consolidation and epithelial hyperplasia is consistent with a shorter time span of lung infection in the contact-infected, rather than the experimentally infected, hamsters.

All RDV-treated animals also exhibited histological changes consistent with SARS-CoV-2 infection, and in the three hamsters where viral antigen expression was seen, they were represented by areas of alveoli with desquamed AEC/AM, as seen in other groups; their extent varied (Supplementary Table S1). In one animal (animal #1), where neither viral RNA nor viral antigen was detected, the histological changes were very mild (widespread evidence of activated type II pneumocytes and occasional syncytial cells; Figure 6F; Supplementary Figure S1C), indicating that this animal was not infected. In the second hamster with viral RNA levels below LOD in the right lung, nasal turbinate, and throat swab (animal #2), moderate viral antigen expression was detected; this animal also showed hyperplasia of type II pneumocytes/BEC, which indicated that (initial) pulmonary infection had occurred at least 3 days earlier [33]. The remaining two hamsters from the group (animals #3 and #4), both found to carry high N-RNA levels, exhibited limited viral antigen expression in the left lung (Supplementary Table S1). The RDV with PIB treatment group was the most heterogeneous. In one animal (animal #4), the histological findings in the left lung did not provide strong evidence of SARS-CoV-2 infection and no viral antigen expression. Together with the viral N-RNA levels below LOD in the right lung, the nasal turbinates, and the throat swab (Figure 6G), this indicates that the hamster had not become infected either (Supplementary Table S1). In the remaining three animals from this group, there was evidence of lung infection, either by PCR and histology (animal #1) or by histology and immunohistology (animal #2) or by all three parameters (animal #3).

Individual animal data are provided in Supplementary Tables S1 and S3.

## 4. Discussion

The presented data describe and evaluate the chemoprophylactic efficacy of PIB, FVP, RDV, FVP with PIB, or RDV with PIB when administered intranasally twice daily to Syrian golden hamsters exposed to SARS-CoV-2 via contact transmission. Activity of the exonuclease complex has been shown to decrease the efficacy of FVP and RDV in vitro [17]. The co-administration of PIB with either FVP or RDV has previously been demonstrated to increase the concentrations of FVP or RDV in vitro [18], which suggests an interesting therapeutic opportunity warranting in vivo evaluation. To test this, PIB was administered in combination with FVP and RDV, and chemoprophylactic efficacy was assessed.

Weight loss is a well-established clinical parameter in SARS-CoV-2-infected rodents [31]. In the present study, hamsters intranasally infected with SARS-CoV-2 had begun to lose weight by 1 dpi, at a time when we did not yet find evidence of viral shedding (qPCR for viral N on throat swabs). At the end of the observation period, at 7 dpi, the animals had started to gain weight again, which can be interpreted as a sign of clinical recovery [34]. The histological findings align with these, showing evidence of pulmonary regeneration, as evidenced by substantial hyperplasia of type II pneumocytes/BEC [33] and very limited viral antigen expression, together with low viral N-RNA levels in most animals.

The saline control group began to lose weight by 3 dpi and continued losing weight until the end of the study. The viral N-RNA levels in throat swabs were below the LOD at day 1, but were positive at day 3, 5, and 7, which would reflect the time window for contact transmission of virus shed by the experimentally infected animal [31]. In all animals of this group, infection of the respiratory tract and lung and virus shedding was confirmed on day 7, through detection of viral N-RNA in nasal turbinate, throat swabs, and lungs; the histological changes and widespread viral antigen expression in the lungs suggest that in the majority, the lungs were infected for at least 3 days [33].

In the PIB treatment group, weight loss was already observed at days 0 and 1, i.e., prior to virus shedding by the infected hamster and hence their potential infection, and continued to be significantly more extensive than in the saline-treated control group until day 5. While this greater weight loss may be attributed to a toxic effect of PIB, a similar weight loss was not observed in the FVP with PIB or RDV with PIB groups, which received the same dose of PIB (20 mg/kg/day). However, while PIB treatment did not avoid infection or shedding (all PCR tests yielded positive results at any examined time point from day 3 onwards), the viral N-RNA levels in the nasal turbinate and lungs were significantly lower than in the saline control group, indicating a mild quantitative chemoprophylactic effect.

Both the FVP and FVP with PIB treatment groups showed progressive weight loss from day 4 onwards, one day after the control group, but without significant quantitative differences. While a few hamsters of these groups tested negative for viral N-RNA in one of the respiratory tract samples, they were all found to harbour SARS-CoV-2 in the lungs at day 7, at slightly, although significantly, lower levels than the saline control group. The histological features suggested that the infection of the lungs had also occurred at least 3 days prior to death in most of these animals. Previous experimental studies have reported high doses of FVP (1400 mg/kg/day) to reduce infectious virus titres when administered intraperitoneally, but these high doses elicited signs of toxicity in hamsters, evidenced by significant weight loss from the first day of treatment [22]. Importantly, even though high doses of FVP were needed for efficacy in previous studies, the plasma concentrations at these high doses were within the range of those observed in humans [35]. Therefore, care should be taken when interpreting preclinical safety in the context of humans due to the evident species differences in exposure and/or response. Despite having used a daily dose 70 times lower (20 vs. 1400 mg/kg), the data presented here show a significant reduction in viral N-RNA in the lungs of both the FVP and FVP with PIB treatment groups, without significant associated weight loss. This apparent disparity could be related to the route of administration used, as the intranasal route would favour a more direct exposure of the target tissues (upper respiratory tract and lungs) to FVP, reducing systemic exposure.

The RDV treatment group showed weight loss on day 0 and, more pronounced, on day 1, when this group’s average weight was significantly lower than that of the saline control group. However, at day 2, the animals regained some weight and then developed weights that were significantly above those of the saline group from day 6 onwards. As for PIB, it is difficult to explain this phenomenon by possible toxic effects of RDV, since the initial downward trend was not observed in the RDV with PIB group, while the progressive weight gain started already on day 4. A drug–drug interaction between RDV and PIB cannot be ruled out. Apart from one animal in the RDV with PIB treatment group, none of the hamsters in these two groups seemed to shed virus at the end of the study, at day 7 (viral N-RNA values below the LOD in nasal turbinate and throat swabs). However, lung infection, detected by PCR and/or immunohistology, with associated histological changes was detected in most (6/8) animals. Nevertheless, in two hamsters, #1 and #4 in the RDV and RDV with PIB treated groups, respectively, infection was not confirmed, as they were negative for all virus detection approaches and only showed minimal histological changes in the lungs that are consistent with, but no proof of, SARS-CoV-2 infection (increased interstitial cellularity, presence of activated type II pneumocytes).

In general, no benefit was observed when combining PIB with FPV or RDV. This observation may be explained by compensatory mechanisms present in vivo that are not recapitulated in vitro. Previous studies showed that the combination of suboptimal doses of FVP (300 mg/kg/day, intraperitoneal) with molnupiravir (nucleoside analogue) resulted in a synergistic reduction in the infectious titres in lungs and nearly completely prevented transmission to co-housed untreated hamsters from infected hamsters treated with the combination [20]. The data presented in the current manuscript describe drug delivery via the intranasal route; this is likely to deliver FVP in a more direct way to the pulmonary parenchyma, i.e., the alveoli, than the intraperitoneal route. However, the lower doses and route of administration used here are extremely unlikely to provide meaningful systemic concentrations of the drugs.

In the case of RDV, a study of formulated RDV (RDV-Leucine; 80/20 *w*/*w*) dry powder for inhalation in hamsters showed that a single dose of the formulation (10 mg/kg) was able to achieve a high effective concentration against SARS-CoV-2 [36]. Unfortunately, differences in formulation make it difficult to draw direct comparisons. Another study evaluated and showed that the combination of RDV (15 mg/kg/day, intraperitoneal) and cobicistat (CYP3A inhibitor) decreases viral replication and disease progression in infected Syrian hamsters [23]. Nevertheless, while the authors showed a synergistic effect between both drugs in vitro, they could not replicate this effect in vivo.

While Syrian golden hamsters have been widely used as a study model for intranasal SARS-CoV-2 infection, species–specific differences to humans must be considered. On one hand, replication of SARS-CoV-2 in hamsters occurs during the first days following infection, with rapid clearance after 3 dpi, whereas in humans, infection of the lung usually lasts much longer [31,37,38,39]. The effectiveness of the treatments studied in this model must be carefully considered since the decrease in viral load by the drug may be accompanied by natural clearance of the virus by the animal. In our case, for a contact transmission study, the experimental design considers the time window in which the experimentally infected animal can spread the virus to the treated animals, which leads to disease of the contact-infected animals by day 3 or later, as confirmed by the histological features. Moreover, hamsters are obligate nasal breathers, which obviates direct extrapolation of the results to humans. Also, the normal respiratory rate for healthy human adults is between 12 and 20 breaths per minute, while hamsters take between 100 and 250 breaths per minute, which could increase the infection rate or success.

Overall, significant reductions in the viral load were observed in nasal turbinate and lungs in the PIB-treated group, whereas significant differences were achieved only in lungs for the FVP and FVP with PIB treatments. In general, only FVP and RDV, alone or in combination with PIB, seem to have any effect on the establishment of infection and the extent of the resulting inflammatory response in the lungs following contact transmission. It was observed that transmission of SARS-CoV-2 was blocked in two animals, one treated intranasally with RDV, another with RDV with PIB, suggesting a putative application of RDV for SARS-CoV-2 chemoprophylaxis via intranasal delivery. No synergistic effect was evident when FVP or RDV were co-administered with PIB.

## Data Availability

The data presented in this study are available upon request from the corresponding author or can be found in Supplementary Tables S1 and S2.

## Supplementary Materials

The following supporting information can be downloaded at: https://www.mdpi.com/article/10.3390/v15112161/s1. Table S1: Relevant histological changes and SARS-CoV-2 nucleoprotein expression in Syrian hamsters after intranasal infection with SARS-CoV-2 B.1.1.7 (Alpha) and euthanised at 7 days post infection, and in hamsters that were exposed to contact transmission for 7 days and treated intranasally with saline, PIB, FVP, FVP+PIB, RDV, or RDV+PIB for 7 days. Table S2: Average viral RNA levels (copies of N-RNA/µg of RNA relative to 18S) in throat swabs (day 1, 3, 5, and 7), nasal turbinate (day 7) and right lung (day 7) for groups treated with saline, PIB, FVP, FVP+PIB, RDV, and RDV+PIB. In parenthesis, the *p* value of each group in comparison to the saline group. Table S3: Individual hamsters exposed to contact transmission for 7 days and treated intranasally with FVP, FVP+PIB, RDV, or RDV+PIB for 7 days that were tested negative for SARS-CoV-2 infection in one or more of the different detection approaches. Figure S1: Closer view of histological changes and viral antigen expression in the lungs of hamsters intranasally infected with SARS-CoV-2 (A) or treated with saline (B) or RDV (C) and housed together as a group of 5 hamsters with an infected hamster.
