# Supplementary material for: Chemoprophylactic Assessment of Combined Intranasal SARS-CoV-2 Polymerase and Exonuclease Inhibition in Syrian Golden Hamsters
Source: Viruses. 2023 Oct 27;15(11):2161. doi: 10.3390/v15112161 (PMC10675045; doi:10.3390/v15112161)
Supplement: Supplementary file 1 [file viruses-15-02161-s001.zip › viruses-2689115-supplementary.pdf]

Supplementary Table S1. Relevant histological changes and SARS-CoV-2 nucleoprotein expression in Syrian hamsters after intranasal infection with 10^4 PFU SARS-CoV-2 B.1.1.7 (Alpha) and euthanised at 7 days post infection ("Infected #1-#6), and in hamsters that were exposed to contact transmission (CT) for 7 days and treated intranasally with saline, PIB, FVP, FVP+PIB, RDV, or RDV+PIB for 7 days (from day -1 to day 6)**.**

NB: In all animals the lungs exhibited a variable degree of increased interstitial cellularity due to leukocyte influx. This is not specifically mentioned in the histological descriptions.

Animal group + no	Treatment, exposure	Histological changes and viral antigen expression (lung)	Virology (PCR)[1]
Infected #1	Infected	**Lung (HE)**: mf to coalescing consolidated areas with type II pc/BEC hyperplasia, leukocyte infiltration[2] and some deg cells; mild pa edema, mild pv LC infiltration<br>**vAg**: a few pos macrophages and very small patches of alveoli with pos AEC	S: 260191<br>N: 19356510<br>L: 105225
Saline #1	Saline;<br>CT from Infected #1	**Lung (HE)**: bronchioles with rare individual deg BEC and mild (sub)epithelial leukocyte infiltration; extensive coalescing areas of alveoli with desquamed AEC/AM, some fibrin, activated type II pc, some syncytial cells and some deg cells, a few LC and NL; mild vascular and pv leukocyte infiltration and pa edema<br>**vAg**: rare pos BEC; multiple large patches of alveoli with pos AEC	S: 71551<br>N: 4588954<br>L: 214215063
Saline #2	Saline;<br>CT from Infected #1	**Lung (HE)**: bronchioles with rare individual deg BEC and mild (sub)epithelial leukocyte infiltration; mf consolidated areas with type II pc/BEC hyperplasia, leukocyte infiltration and some deg cells; mf coalescing areas of alveoli with desquamed AEC/AM, some fibrin, activated type II pc, some syncytial cells and some deg cells, a few LC and NL; mild vascular infiltration and pa edema<br>**vAg**: rare pos BEC; multiple large patches of alveoli with pos AEC	S: 925835<br>N: 13001195<br>L: 255314234
Saline #3	Saline;<br>CT from Infected #1	**Lung (HE)**: mf areas of alveoli with desquamed AEC/AM, alveolar edema, activated type II pc, some syncytial cells and some deg cells; other areas with activated type II pc, a few syncytial cells, macrophages, LC and deg cells; mild to mod vascular leukocyte infiltration<br>**vAg**: multiple large patches of alveoli with pos AEC	S: 63743657<br>N: 11071723<br>L: 130573481
Saline #4	Saline;<br>CT from Infected #1	**Lung (HE)**: mf coalescing areas of alveoli with desquamed AEC/AM, some fibrin, activated type II pc, some syncytial cells, some deg cells, a few LC and NL; small consolidated areas with activated type II pc and focal type II pc/BEC hyperplasia; mild vascular infiltration and pa edema<br>**vAg**: rare pos BEC; multiple large patches of alveoli with pos AEC	S: 64771<br>N: 13931410<br>L: 120151650
Saline #5	Saline;<br>CT from Infected #1	**Lung (HE)**: mf coalescing areas of alveoli with desquamed AEC/AM, some fibrin, activated type II pc, some syncytial cells, some deg cells, a few LC and NL; mf consolidated areas with activated type II pc and type II pc/BEC hyperplasia; mild vascular infiltration and pa edema<br>**vAg**: small patches of alveoli with pos AEC	S: 166482<br>N: 71845150<br>L: 130309340
Infected #2	Infected	**Lung (HE)**: mf to coalescing consolidated areas with with activated type II pc and type II pc/BEC hyperplasia, a few syncytial cells, mod numbers of macrophages and a few NL and deg cells; mild to mod vascular infiltration and pa edema; mild pv LC infiltration<br>**vAg**: rare individual and small patches of alveoli with pos AEC	S: 53452<br>N: 1553459<br>L: 110368
PIB #1	Pibrentasvir;<br>CT from Infected #2	**Lung (HE)**: mf to coalescing consolidated areas with focal type II pc/BEC hyperplasia, macrophages, a few NL and deg cells, focal areas with desquamation of AEC/AM and some syncytial cells; mild to mod leukocyte emigration and pv infiltration<br>**vAg**: numerous small patches of alveoli with pos AEC (within and outside areas of desquamation)	S: 58937939<br>N: 240222<br>L: 33935538
PIB #2	Pibrentasvir;<br>CT from Infected #2	**Lung (HE)**: mf, mainly loosely consolidated areas with activated type II pc and focal type II pc/BEC hyperplasia, mod numbers of macrophages, a few NL and deg cells; mild to mod vascular infiltration, mild pv LC infiltration<br>**vAg**: abundant variably sized patches of alveoli with pos AEC (outside, in the periphery of and within consolidated areas)	S: 12774<br>N: 1496389<br>L: 21571524

PIB #3	Pibrentasvir; CT from Infected #2	**Lung (HE)**: mf to coalescing consolidated areas with type II pc/BEC hyperplasia, macrophages, a few NL and deg cells, focal areas with desquamation of AEC/AM and some syncytial cells; mild to mod vascular and pv LC infiltration, mild pa edema<br>**vAg**: abundant variably sized patches of alveoli with pos AEC (outside, in the periphery of and within consolidated areas)	S: 75551<br>N: 10667159<br>L: 32116347
PIB #4	Pibrentasvir; CT from Infected #2	**Lung (HE)**: mf to coalescing consolidated areas with type II pc/BEC hyperplasia, macrophages, a few NL and deg cells, focal areas with desquamation of AEC/AM and some syncytial cells; mild to mod vascular and pv LC infiltration, mild pa edema<br>**vAg**: extensive, mainly large patches of alveoli with pos AEC (outside and in periphery of consolidated areas)	S: 58071<br>N: 9445262<br>L: 16032641
Infected #3	Infected	**Lung (HE)**: mf to coalescing consolidated areas with type II pc/BEC hyperplasia, leukocyte infiltration and some deg cells; mod pa edema; mild pv LC-dom leukocyte infiltration<br>**vAg**: a few individual pos AEC and macrophages in consolidated areas, several small disseminated patches of alveoli with pos AEC	S: 50238183<br>N: 4545371<br>L: 110021
FVP #1	Favipiravir; CT from Infected #3	**Lung (HE)**: mf areas with desquamation of AEC/AM, macrophages, NL, some syncytical cells and some deg cells; mild to mod LC-dom arteritis and pa infiltration<br>**vAg**: rare individual intact pos BEC; extensive large patches of alveoli with pos AEC	S: 717120<br>N: <LOD<br>L: 45555269
FVP #2	Favipiravir; CT from Infected #3	**Lung (HE)**: bronchioles with individual deg EC; mf areas with desquamation of AEC/AM, macrophages, NL, some syncytical cells and some deg cells; focal areas of type II pc/BEC hyperplasia; mild to mod LC-dom vascular and pa infiltration<br>**vAg**: rare individual intact and deg pos BEC; mf disseminated small to large patches of alveoli with pos AEC	S: <LOD<br>N: 16107403<br>L: 105791946
FVP #3	Favipiravir; CT from Infected #3	**Lung (HE)**: very widespread desquamation of AEC/AM, with fibrin, macrophages, NL, some syncytical cells and some deg cells in alveoli, alveolar edema and extensive alveolar hemorrhage; mild pv LC-dom infiltration<br>**vAg**: rare individual intact and deg pos BEC; very widespread disseminated and coalescing large patches of alveoli with pos AEC	S: 44627<br>N: 9746003<br>L: 66193065
FVP #4	Favipiravir; CT from Infected #3	**Lung (HE)**: very widespread desquamation of AEC/AM, with fibrin, macrophages, NL, some syncytical cells and some deg cells in alveoli, alveolar edema and mf alveolar hemorrhage; focal consolidated areas with type II pc/BEC hyperplasia and leukocyte infiltration; mild pv LC-dom infiltration<br>**vAg**: mf disseminated variably sized patches of alveoli with pos AEC	S: <LOD<br>N: 23753666<br>L: 55222081
Infected #4	Infected	**Lung (HE)**: mf to coalescing consolidated areas with type II pc/BEC hyperplasia, leukocyte infiltration and some deg cells; mod pa edema; mild pv LC-dom leukocyte infiltration<br>**vAg**: a few individual pos AEC and macrophages in consolidated areas, a few mod sized disseminated patches of alveoli with pos AEC	S: 20110<br>N: 8413686<br>L: 160505
FVP+PIB #1	Favipiravir and Pibrentasvir; CT from Infected #4	**Lung (HE)**: mf areas with desquamation of AEC/AM, some deg cells, occ syncytial cells, some leukocytes; small consolidated areas with type II pc/BEC hyperplasia, leukocyte infiltration and some deg cells; mild to mod pv LC-dom leukocyte infiltration<br>**vAg**: many variably sized disseminated patches of alveoli with pos AEC	S: 2268658<br>N: 2941343<br>L: 29975435
FVP+PIB #2	Favipiravir and Pibrentasvir; CT from Infected #4	**Lung (HE)**: bronchioles with occ deg EC and desquamed EC in lumen, mild (sub)epithelial LC-dom infiltration; mf areas with desquamation of AEC/AM, some deg cells, occ syncytial cells, some leukocytes; small consolidated area with type II pc/BEC hyperplasia, leukocyte infiltration and some deg cells; mild to mod pv LC-dom leukocyte infiltration<br>**vAg**: most bronchioles with several individual and patches of intact pos EC; several variably sized patches of alveoli with pos AEC	S: 36979666<br>N: 4151342<br>L: 107999927

FVP+PIB #3	Favipiravir and Pibrentasvir; CT from Infected #4	**Lung (HE)**: mf to coalescing consolidated areas with type II pc/BEC hyperplasia, leukocyte infiltration and some deg cells; some areas with desquamation of AEC/AM, some deg cells, occ syncytial cells, some leukocytes; mild pa edema; mild vascular and pv LC-dom leukocyte infiltration<br>**vAg**: a few individual pos AEC and macrophages in consolidated areas, widespread large patches of alveoli with pos AEC	S: 103771<br>N: 14027330<br>L: <LOD
FVP+PIB #4	Favipiravir and Pibrentasvir; CT from Infected #4	**Lung (HE)**: mf to coalescing consolidated areas with type II pc/BEC hyperplasia, leukocyte infiltration and some deg cells; some areas with desquamation of AEC/AM, some deg cells, occ syncytial cells, some leukocytes; mild pa edema; mild vascular and pv LC-dom leukocyte infiltration<br>**vAg**: a few individual pos AEC and macrophages in consolidated areas, a few disseminated mod sized patches of alveoli with pos AEC	S: 477534<br>N: 28217555<br>L: 56590693
Infected #5	Infected	**Lung (HE)**: mf to coalescing consolidated areas with activated type II pc and type II pc/BEC hyperplasia, a few syncytial cells, mod numbers of macrophages, a few NL and deg cells; focal alveolar edema; mild to mod vascular infiltration and pa edema; mild pv LC-dom infiltration<br>**vAg**: a few individual and small patches of alveoli with pos AEC	S: 42575<br>N: 43140212511<br>L: 8736570121
RDV #1	Remdesivir; CT from Infected #5	**Lung (HE)**: widespread activated type II pc; occ syncytial cells<br>**vAg**: neg	S: <LOD<br>N: <LOD<br>L: <LOD
RDV #2	Remdesivir; CT from Infected #5	**Lung (HE)**: mf extensive areas with desquamation of AEC/AM, macrophages, NL and deg cells, with mild focal type II pc/BEC hyperplasia; very mild (peri)bronchial LC infiltration; mod LC-dom vascular and pv infiltration<br>**vAg**: a few individual pos intact BEC; several variably sized patches of alveoli with pos AEC	S: <LOD<br>N: <LOD<br>L: <LOD
RDV #3	Remdesivir; CT from Infected #5	**Lung (HE)**: focal areas with desquamation of AEC/AM, activated type II pc, NL and deg cells; mild (peri)arterial LC-dom infiltration<br>**vAg**: several bronchioles with a few to numerous intact pos EC and a few pos deg cells in lumen; one focal area of alveoli with pos AEC	S: <LOD<br>N: <LOD<br>L: 201109102313
RDV #4	Remdesivir; CT from Infected #5	**Lung (HE)**: focal subpleural area with desquamation of AEC/AM, macrophages, NL and deg cells; small focal consolidated area with type II pc/BEC hyperplasia, a few deg cells and a few NL; mild (peri)arterial LC infiltration<br>**vAg**: one large area (desquamative lesion) of alveoli with pos AEC	S: <LOD<br>N: <LOD<br>L: 25667286385
Infected #6	Infected	**Lung (HE)**: mf to coalescing consolidated areas with activated type II pc and type II pc/BEC hyperplasia, focal desquamation of AEC/AM, a few syncytial cells, mod numbers of macrophages, a few NL and deg cells; mild to mod LC-dom arteritis and pa infiltration, mod pa edema; mild pv LC infiltration<br>**vAg**: a few small patches of alveoli with pos AEC	S: 111238<br>N: 1707945<br>L: <LOD
RDV+PIB #1	Remdesivir and Pibrentasvir; CT from Infected #6	**Lung (HE)**: a few small focal areas with desquamation of AEC/AM, leukocyte infiltration, activated type II pc and occ deg cells<br>**vAg**: neg	S: <LOD<br>N: <LOD<br>L: 22827806270
RDV+PIB #2	Remdesivir and Pibrentasvir; CT from Infected #6	**Lungs**: focal consolidated areas with activated type II pc and type II pc/BEC hyperplasia, occ syncytial cells, macrophages and LC, occ deg cells; focal areas with desquamation of AEC/AM, leukocyte infiltration and activated type II pc; mild to mod pb LC-dom infiltration<br>**vAg**: very rare individual pos intact BEC and AEC	S: <LOD<br>N: <LOD<br>L: <LOD
RDV+PIB #3	Remdesivir and Pibrentasvir; CT from Infected #6	**Lung (HE)**: focal consolidated area with activated type II pc and type II pc/BEC hyperplasia, occ syncytial cells, macrophages and LC, occ deg cells; smaller focal areas with desquamation of AEC/AM, leukocyte infiltration and activated type II pc; mod pb LC-dom infiltration<br>**vAg**: several bronchioles with several to numerous pos EC	S: 848238<br>N: 523848409877<br>L: 28732905109

RDV+PIB #4	Remdesivir and Pibrentasvir; CT from Infected #6	**Lung (HE)**: multifocal activated type II pc; random small macrophage aggregates<br>**vAg**: neg	S: <LOD<br>N: <LOD<br>L: <LOD

Legend: AEC – alveolar epithelial cells; AM – alveolar macrophages; BEC – bronchiolar epithelial cells; deg – degenerate; EC – epithelial cells; FVP – Favipiravir; HE – histological features assessed in a hematoxylin-eosin stained section; LC – lymphocyte; mf – multifocal; mod – moderate; neg – negative; NL – neutrophils; NT – nasal turbinates; LC – lymphocytes; LC-dom – lymphocyte dominated; LOD – level of detection; occ – occasional; pb – peribronchiolar; pc – pneumocytes; PF – proteinaceous fluid; PIB – Pibrentasvir; pos – positive; pv – perivascular; RDV – Remdesivir; vAg – viral antigen

[1] PCR: Copies of viral N-RNA/µg of RNA relative to 18S, for each animal determined in throat swabs (S), nasal turbinates (N) and lungs (L) at day 7.

[2] leukocyte infiltration/leukocyte infiltrates: if not further specified, this implies macrophages, fewer lymphocytes and variable numbers of neutrophils

Supplemental Table S2. Average viral RNA levels (copies of N-RNA/µg of RNA relative to 18S) in throat swabs (day 1, 3, 5, and 7), nasal turbinate (day 7) and right lung (day 7) for groups treated with saline, PIB, FVP, FVP+PIB, RDV, and RDV+PIB. In parenthesis, the p value of each group in comparison to the saline group. Values significantly lower than in the saline group are shown in bold (* = P ≤ 0.05, ** = P ≤ 0.01, nonparametric Mann-Whitney test, one-tailed).

Group	Throat swabs				Nasal turbinate (day 7)	Right lung (day 7)
	Day 1	Day 3	Day 5	Day 7		
Saline	<LOD	7.3x10^{7}	4.3x10^{8}	1.3x10^{7}	2.3x10^{7}	1.7x10^{8}
PIB	<LOD<br>(P = 0.5)	1.9x10^{7}<br>(P = 0.365)	1.5x10^{8}<br>(P = 0.365)	1.5x10^{7}<br>(P = 0.206)	**5.5x10^{6}<br>(*P = 0.032)**	**2.6x10^{7}<br>(**P = 0.008)**
FVP	<LOD<br>(P = 0.5)	8.4x10^{6}<br>(P = 0.143)	2.1x10^{7}<br>(P = 0.278)	**1.9x10^{5}<br>(*P = 0.048)**	1.2x10^{7}<br>(P = 0.452)	**6.8x10^{7}<br>(**P = 0.008)**
FVP+PIB	<LOD<br>(P = 0.5)	6.5x10^{8}<br>(P = 0.365)	5.4x10^{8}<br>(P = 0.278)	9.9x10^{6}<br>(P = 0.279)	1.2x10^{7}<br>(P = 0.365)	**4.9x10^{7}<br>(**P = 0.008)**
RDV	<LOD<br>(P = 0.5)	**<LOD<br>(**P = 0.008)**	**<LOD<br>(**P = 0.008)**	**<LOD<br>(**P = 0.008)**	**<LOD<br>(**P = 0.008)**	5.7x10^{10}<br>(P = 0.5)
RDV+PIB	<LOD<br>(P = 0.5)	**<LOD<br>(**P = 0.008)**	**2.2x10^{5}<br>(*P = 0.016)**	2.1x10^{5}<br>(P = 0.056)	1.3x10^{11}<br>(P = 0.143)	1.3x10^{10}<br>(P = 0.5)

Supplementary Table S3. Individual hamsters exposed to contact transmission (CT) for 7 days and treated intranasally with FVP, FVP+PIB, RDV, or RDV+PIB for 7 days (from day -1 to day 6) that were tested negative for SARS-CoV-2 infection in one or more of the different detection approaches. NB: All saline and PIB treated hamsters tested positive in all assays.

**Treatment**	**Animal no (#)**	**PCR[1]**			***In situ*[2]**	
		**Tracheal swab**	**Nasal turbinate**	**Lung**	**IH (vNP)**	**Histology**
FVP	1	+	-	+	+	+
	2	-	+	+	+	+
	4	-	+	+	+	+
FVP+PIB	3	+	-	-	+	+
RDV	**1[α, γ]**	**-**	**-**	**-**	**-**	(+)
	2[α, β]	-	-	-	+	+
	3[α, β]	-	-	+	+	+
	4[α, β]	-	-	+	+	+
RDV+PIB	1[α, β]	-	-	+	-	(+)
	2[α, β]	-	-	-	(+)	+
	**4[α, γ]**	**-**	**-**	**-**	**-**	(+)

Legend: FVP – Favipiravir; IH - immunohistology; PIB – Pibrentasvir; RDV – Remdesivir; vNP – viral, i.e. SARS-CoV-2 nucleoprotein

[1] PCR: Copies of viral N-RNA/µg of RNA relative to 18S, for each animal determined in throat swabs (S), nasal turbinates (N) and lungs (L) at day 7.

[2] *In situ*: detection of viral antigen by immunohistology (IH) or histological changes consistent with SARS-CoV-2 infection (consolidated areas with focal hyperplasia of type II pneumocytes/bronchial epithelial cells and/or areas of alveoli with desquamed alveolar epithelial cells/alveolar macrophages, presence of activated type II pneumocytes, evidence of syncytial cells and of degenerate cells). Grading for IH: - negative (no viral antigen detected); (+) very rare individual positive alveolar and/bronchiolar epithelial cells; + variable amount of alveoli with positive alveolar epithelial cells (and positive bronchiolar epithelial cells). Grading for Histology: (+) minimal changes consistent with SARS-CoV-2 infection (increased interstitial cellularity, presence of activated type II pneumocytes; + changes consistent with SARS-CoV-2 infection, as defined above.

[α] No evidence of viral shedding at the time of death.

[β] Evidence of pulmonary infection (PCR, IH and/or histology).

[γ] Infection of animal not confirmed.

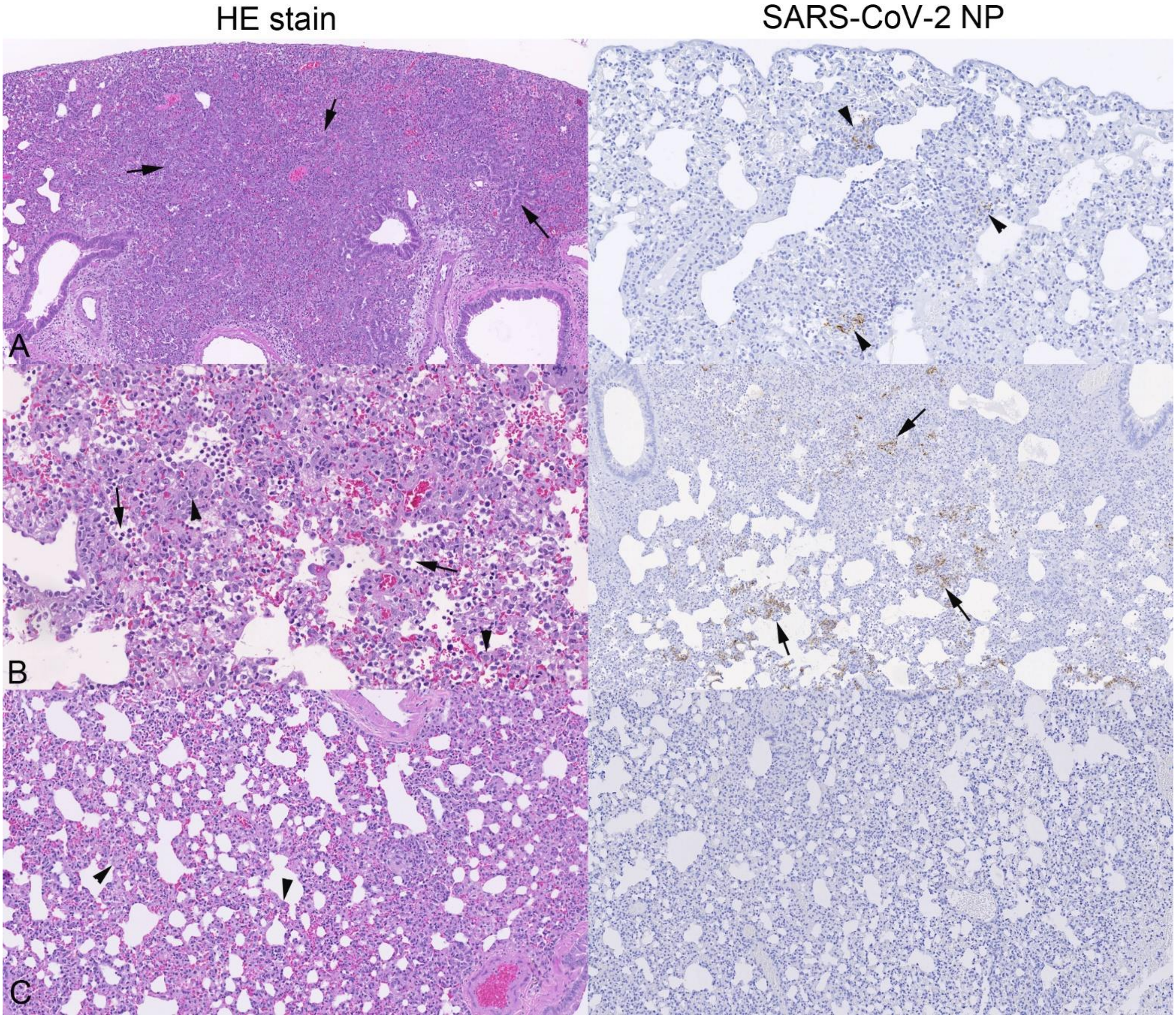

Supplementary Figure S1. Closer view of histological changes and viral antigen expression in the lungs of hamsters intranasally infected with SARS-CoV-2 (A) or treated with saline (B) or RDV (C) and housed together as a group of 5 hamsters with an infected hamster; animals were euthanized and examined at day 7. Left column: HE stained sections, right column: consecutive sections stain for SARS-CoV-2 nucleoprotein (NP), immunohistology, hematoxylin counterstain. **A.** Infected hamster (#3). Consolidated area (corresponding to area indicated by the arrow in Figure 6A) with multifocal type II pneumocytes/bronchiolar epithelial hyperplasia (arrows). Viral antigen expression is seen in a few alveolar epithelial cells (AEC) and macrophages (arrowheads). **B.** Saline treated hamster (#4). Focal area (corresponding to area indicated by the arrow in Figure 6B) of alveoli that contain desquamed AEC and/or alveolar macrophages (arrows) and exhibit activated type II pneumocytes (arrowheads). Viral antigen expression is seen in AEC in multiple patches of alveoli (arrows). **C.** RDV treated hamster (#1). **C.** RDV treated hamster (#1). The parenchyma appears cell rich, consistent with increased interstitial cellularity. There are activated type II pneumocytes (arrowheads). There is no evidence of viral antigen expression (right image).
